# Association of Epstein-Barr virus (EBV) with lung cancer: meta-analysis

**DOI:** 10.3389/fonc.2023.1177521

**Published:** 2023-10-04

**Authors:** Yizhuo Chen, Tianhua Liu, Ziqing Xu, Ming Dong

**Affiliations:** Department of Lung Cancer Surgery, Tianjin Medical University General Hospital, Tianjin, China

**Keywords:** Epstein-Barr virus, lung cancer risk, carcinogenesis, lung cancer, lung tumor

## Abstract

**Objective:**

Epstein-Barr virus (EBV) is a virus that is ubiquitous in humans. To investigate the association between EBV infection and lung cancer risk to reveal whether it is involved in the development and development of lung cancer. Although there has been discussion of EBV and lung cancer in the past. Through this study, we hope to deepen our understanding of the causes of lung cancer and provide new clues and targets for the prevention, early diagnosis and treatment of lung cancer. This study is also beneficial to the development of medical science and public health. First of all, the research results are expected to be incorporated into lung cancer prevention and treatment strategies and policies, so as to provide better treatment decisions for lung cancer patients and improve the survival rate and quality of life of patients. At the same time, communicating the research results to the public can help raise awareness of lung cancer risk factors. By encouraging healthy lifestyles and screening measures, the public can reduce their risk of lung cancer. In addition, this study also provides an important foundation for subsequent academic research and scientific exploration. It provides valuable information and inspiration for in-depth understanding of lung cancer and other related fields. Overall, this study makes an important contribution to both medical science and public health.

**Method:**

By September 26, 2022, an online database was used to conduct a literature search in English. Random effects models were employed to estimate the prevalence of EBV with 95% confidence intervals (CIs). Additionally, the pooled odds ratio (OR) and 95%CI were calculated from case-control studies to determine the association between EBV and lung cancer.

**Results:**

In this study of 886 patients with lung cancer, the overall prevalence of EBV infection was found to be 44.36% (95%CI: 4.08-16.9). Fourteen studies were included in the analysis, all of which used a case-control design and involved comparisons of tumors with adjacent or non-adjacent normal and non-cancerous controls. There was a significant difference in the prevalence of EBV infection in lung cancer tissues between China and other regions, with an odds ratio (OR) of 9.36 (95% confidence interval: 4.00-21.94, P<0.001, I²=73.5%). This suggests that the association between EBV infection and lung cancer cases is stronger in China than in other regions. Additionally, the prevalence of EBV infection varied across different pathological types of lung cancer, with rates of 81.08% for pulmonary lymphoepithelioma-like carcinoma (LELC),this a rare subtype of non-small cell lung cancer (NSCLC).34.78% for non-small cell lung cancer, and 21.17% for small cell lung cancer. The statistical analysis indicated that EBV infection was most significantly associated with cancer risk in LELC, while non-small cell lung cancer was more strongly associated with EBV than small cell lung cancer.

**Conclusion:**

The study found that EBV infection increases the risk of lung cancer by more than four times, and this risk is associated with the pathological type, lymphatic infiltration, and degree of differentiation of the lung cancer, particularly in the rare subtype of pulmonary lymphoepithelioma in non-small cell lung cancer(NSCLC). Additionally, there are racial and regional differences in the correlation between EBV-infected lung cancer, with the Asian population showing greater susceptibility. The study used normal or abnormal tissue adjacent to the tumor as a control, which is considered a more accurate method for determining the relationship between EBV infection and lung cancer.

## 1 Introduction

Globally and in China, lung cancer (LC) is a prevalent cause of cancer morbidity and mortality. The development of lung cancer is considered to be multifactorial, involve genetic, environmental, and lifestyle risk factors (1). The development of lung cancer is thought to be influenced by various factors, including smoking, air pollution, occupational factors, genetic factors, biological factors, and ionizing radiation (2). Research studies such as candidate gene studies and genome-wide association studies have identified numerous dysfunctional genes that may be associated with the development of lung cancer. However, these identified susceptibility factors cannot fully account for the observed incidence of lung cancer, indicating that other factors also have a direct impact on lung cancer risk.

Over the years, the role of viruses in the development of cancer has garnered increasing attention among the many known carcinogenic factors (2). The Epstein-Barr virus (EBV) was the first tumor virus to be linked with the development of human malignancies. Research has confirmed that this virus is primarily associated with the pathogenesis of Burkitt lymphoma and nasopharyngeal carcinoma (3).

The respiratory tract serves as a significant reservoir for the Epstein-Barr virus, yet there has been limited investigation into whether an association exists between EBV infection and the development of lung cancer (4). In 1987, Begin reported the first case demonstrating a link between pulmonary lymphoepithelioma-like carcinoma (LELC) and the Epstein-Barr virus (EBV) (5). According to the World Health Organization (WHO) 2015 histological classification, there is an extremely rare subtype of unclassified lung cancer called Pulmonary lymphoepithelioma-like carcinoma (LELC), which was once classified as non-small cell lung cancer (NSCLC). often accompanied by Epstein-Barr virus infection (6), but its prognosis is better than other types of lung cancer (7). Apart from the relatively rare LELC subtype, there is mounting evidence that the Epstein-Barr virus (EBV) is also detectable in tumor cells of the more common subtypes of non-small cell lung cancer (NSCLC), such as lung squamous cell carcinoma (LUSC) and lung adenocarcinoma (LUAD) (8). There is a suggestion that the Epstein-Barr virus (EBV) (9) may have a significant role in the development of lung cancer.

## 2 Methods

### 2.1 Search strategy

We conducted a search in the MeSH and free text fields for the terms “Epstein-Barr Virus,” “EBV,” “Epstein-Barr virus Infections,” or “Human Herpesvirus 4 Infections,” in combination with “lung cancer,” “Lung Neoplasms,” or “Pulmonary Neoplasm.” The search was carried out on various databases including Pubmed, Web of Science, Cochrane Library, and Embase, for publications in English. Additionally, we searched CNKI, National Science and Technology Books and Literature Center (NSTL), Wanfang Data, VIP network, and X-MOL academic platform. Furthermore, we reviewed other relevant references cited in the retrieved articles.

This review follows the PRISMA protocol for systematic reviews and meta-analyses (http://www.prisma-statement.org/). Ethics committee approval is not required.

### 2.2 Research selection

Two authors (CYZ and DM) independently reviewed the titles and abstracts of all relevant studies to assess their eligibility. Studies were included if they met the following criteria: (1) Investigated the association between EBV infection and lung cancer patients by assessing the expression levels of EBV (DNA or antibodies) in tissue samples; (2) Confirmed histopathological diagnosis of lung cancer cases; (3) Reported raw data; (4) Utilized a case-control study design; (5) Employed fresh, frozen, or paraffin-embedded (PE) blood sample storage methods; (6) Used polymerase chain reaction (PCR), reverse transcription PCR (RT-PCR), real-time quantitative PCR (qPCR), *in situ* hybridization (ISH), or immunohistochemistry (IHC) techniques; (7) Had full-text articles available in English or Chinese. Studies that were excluded included case series, reports, animal models, *in vitro* studies, reviews, editorials, conference abstracts, and letters without sufficient data. If multiple publications reported results based on the same study, the more recent article or the one with a larger sample size was included.

### 2.3 Data extraction

The data extraction process was carried out by two authors (CYZ and DM) independently, utilizing a pre-designed data extraction form based on meta-analysis guidelines. Any discrepancies were resolved by discussion or consultation with a third author (LTH). The extracted information included the author’s name, publication year, study location, number of lung cancer cases and controls, sample size, tissue type, assay and markers used, and type of control.

### 2.4 Quality assessment

The Newcastle-Ottawa Scale (NOS) was used to evaluate the methodological quality of the included studies (10), where five stars indicate moderate to high quality.

### 2.5 Statistical methods

When there was sufficient data available, we conducted a meta-analysis and calculated odds ratios (ORs) with corresponding 95% confidence intervals. We checked for heterogeneity between studies by calculating P-values using Cochran’s Q test and the I^2^ statistic. If I^2^ <50%, we used a fixed-effects model (Mantel-Haenszel method) to assess heterogeneity between studies. Otherwise, we used a random-effects model (De Simon and Laird method). We used the Z-test to determine the pooled OR and 95% CI. If necessary, we conducted meta-regression analyses to identify possible sources of heterogeneity. We also conducted subgroup analyses for race and different lung cancer histological subtypes (squamous cell carcinoma and adenocarcinoma).

To evaluate the potential impact of individual studies on the overall risk of lung cancer, a sensitivity analysis was conducted by excluding one study at a time and recalculating the pooled OR and 95% confidence interval. The possibility of publication bias was evaluated visually using a funnel plot proposed by Berger and statistically using Egger’s linear regression test.

All statistical tests were performed using Stata 17.0 (StataCorporation, College Station, TX, USA). P<0.05 was considered statistically significant.

## 3 Results

### 3.1 Literature selection

**Figure 1:**
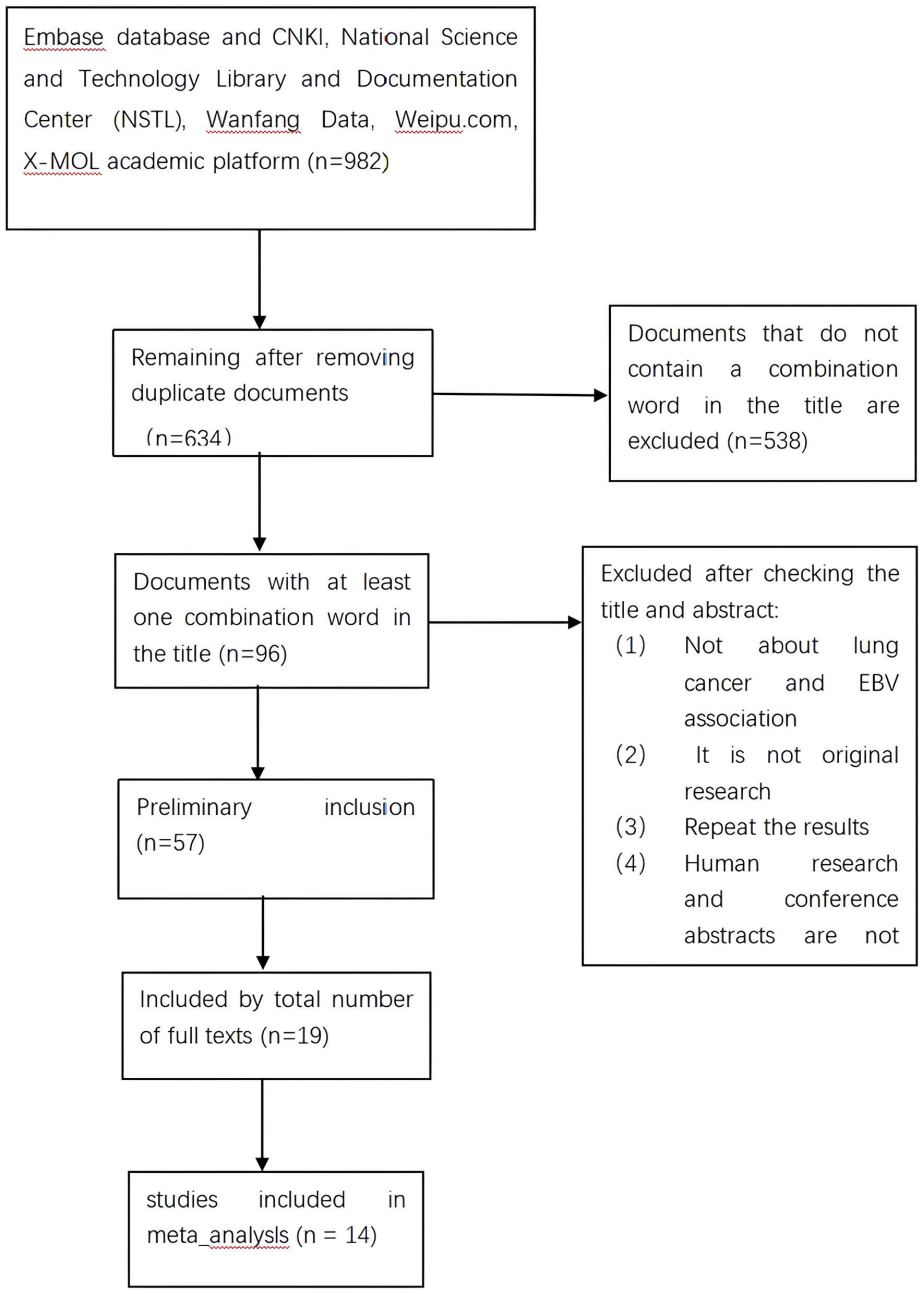
Flow chart of research selection process and literature search results.

Initially, a search of electronic databases identified 982 articles, of which 348 duplicate articles were excluded. After screening titles, 538 articles were further excluded due to their lack of relevance. The remaining 96 articles underwent full-text review, and 39 articles were excluded based on their lack of relevance, leaving 57 articles for further evaluation. After applying inclusion and exclusion criteria, 19 papers were deemed eligible for this systematic review and meta-analysis. Five papers were subsequently excluded based on not meeting meta-analysis criteria, resulting in 14 papers for final inclusion. The search and screening process is illustrated in **Figure 1**.

### 3.2 Study characteristics

**Table 1** lists the characteristics of the fourteen eligible studies, including country of origin, specimen type, EBV detection method, histological type, and number of LC subjects and non-cancer controls with EBV. Among them, ten are carried out in China (1–4, 8, 11–15), and the remaining four are Two were performed in Spain (16, 17), one in the United States (18) and one in Italy (19). Specimens included paraffin-embedded tissue, frozen biopsy, and serum. In addition, seven studies used polymerase chain reaction (PCR)-based techniques to detect EBV (3, 8, 11, 12, 15, 17, 19), and four studies used *In situ* hybridization (ISH) (1, 2, 13, 18), one study detected EBV by polymerase chain reaction (PCR) technology and *in situ* hybridization (ISH) together (16), One study detected EBV by polymerase chain reaction (PCR) technology and enzyme-linked immunosorbent assay (ELISA) (4), and one study used indirect fluorescent immunoassay (IFA) (14). The different types of lung cancer tissue that can be identified through histological analysis include squamous cell carcinoma (SCC), adenocarcinoma (AC), small cell lung cancer (SCLC), large cell lung cancer (LCC), and pulmonary lymphoepithelioma-like carcinoma (LELC). In order to examine the potential relationship between Epstein-Barr virus (EBV) infection and the risk of developing lung cancer, fourteen studies were conducted using both case and control tissues. The control tissues used in these studies included samples taken from patients with pneumothorax, rib fractures, tuberculosis, chest wall deformity, cryptococcal infection, fibrosis, pseudotumor, bullae, pulmonary cyst, chronic obstructive pulmonary disease with bronchial hyperplasia and squamous metaplasia, sarcoidosis, bronchopneumonia, and hamartoma. In total, the fourteen studies analyzed 1,569 tissue samples, consisting of 886 cases and 683 controls.

**Table 1:** Research abstracts included in the meta-analysis.

permutation	First author(Year)	Study location/Specimen type	Inspection technology	Target	Cases of lung cancer (n=886)		Compare cases (n=683)	
					masculine	feminine	masculine	feminine
1	Jose Javier Gómez-Román (2009)	Spain/Paraffin-embedded tissue	Polymerase chain reaction (PCR)/*In situ* hybridization technique for chromogenic	EBV DNA sequence/EBER	12	7	0	90
2	A J Han (2000)	China/Paraffin-embedded tissue	*In situ* hybridization technique for chromogenic	EBER	30	2	0	19
3	Mahdi Karimi-Shahri (2013)	Spain/Paraffin-embedded tissue	Polymerase chain reaction (PCR)	EBV DNA sequence	5	43	2	40
4	Giovanna E. (2018)	Italy/(Bronchial brushes/EBC)	Polymerase chain reaction (PCR)	EBV DNA sequence	40	30	13	27
5	Xia He Shun (2000)	China/Paraffin-embedded tissue	Polymerase chain reaction (PCR)	EBV DNA sequence	25	23	15	17
6	Wang Mei Mei (2013)	China/Paraffin-embedded tissue	*In situ* hybridization technique for chromogenic	EBER1	36	72	1	22
7	Lai Ya Zhou (2005)	China/(Blood samples/Fresh frozen tissue)	Polymerase chain reaction (PCR)	EBV DNA sequence	36	24	6	52
8	Jiang Bo (2016)	China/Fresh frozen tissue	Polymerase chain reaction (PCR)	EBV DNA sequence	11	19	0	30
9	Lai Ya Zhou (2003)	China/Fresh frozen tissue	Polymerase chain reaction (PCR)	EBV DNA sequence	27	33	4	56
10	Zhang Lei (1996)	China/Paraffin-embedded tissue	*In situ* hybridization technique for chromogenic	Epstein-Barr virus cells	33	54	10	77
11	Ma Li Ren (2009)	China/Blood samples	Indirect fluorescence immunoassay (IFA)	Epstein-Barr virus antibodies	73	29	16	16
12	Sun Ya Li (2019)	China/Paraffin-embedded tissue	Polymerase chain reaction (PCR)	EBV DNA sequence	40	32	0	20
13	Kheir (2019)	American/Paraffin-embedded tissue	*In situ* hybridization technique for chromogenic	EBER	3	107	0	110
14	Dong Xue Feng (2007)	China/Fresh frozen tissue	Polymerase chain reaction (PCR)	EBV DNA sequence	17	23	0	40
Total combined volume					388	498	67	616
Prevalence					43.79%		9.81%	

### 3.3 Meta-analysis

**Figure 2:**
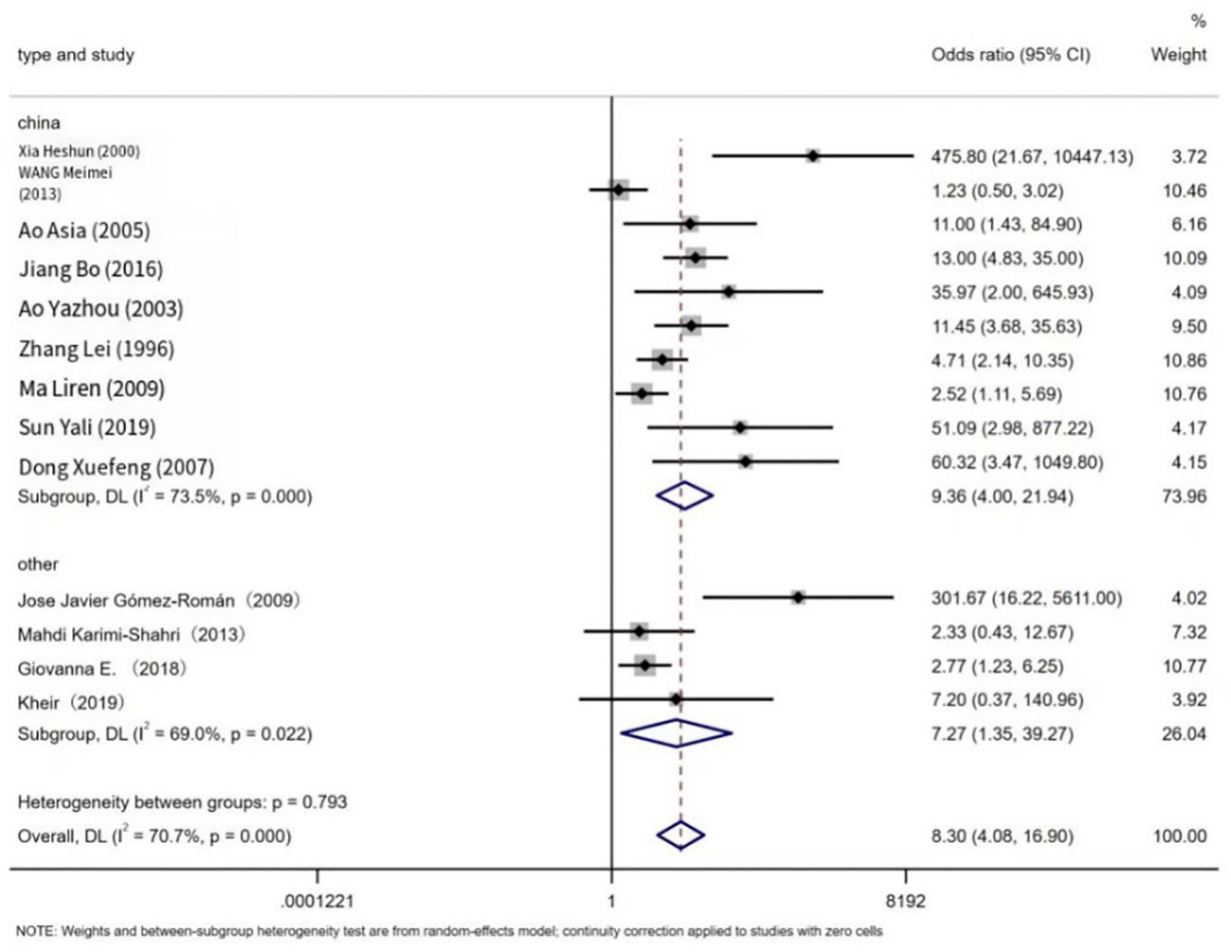
Overall association between EBV infection and lung cancer risk, stratified by race. For each study, estimates of odds ratios (ORs) and their 95% confidence intervals (CIs) are plotted with boxes and horizontal lines. The symbol diamond represents the pooled OR and its 95% confidence interval.

The prevalence of EBV in LC patients was higher than that in non-cancer controls (43.79%, 9.81%). **Figure 2** below shows a forest plot of the overall association between subjects with/without EBV stratified by race. A statistical association was observed between EBV and LC patients (OR = 8.30, 95% CI: 4.08–16.9, P < 0.001; I² = 70.7%). In addition, the significance of the ten Chinese studies was also tested separately (OR = 9.36, 95% confidence interval: 4.00–21.94, P < 0.001, I² = 73.5%) and four other studies (OR = 7.27, 95% CI: 1.35–39.27, P = 0.001, I² = 69.0%). The prevalence of EBV in LC patients in China (Mainland China and Taiwan) reached 41.98%, and the study showed a higher prevalence than that of other ethnic groups.

We also evaluated the cancer risk of EBV in different LC histological types, see **Table 2**. Pulmonary lymphoepithelioma-like carcinoma (LELC) is a subtype of non-small cell lung cancer(NSCLC), but it is relatively rare, so it is compared with common non-small cell lung cancer(NSCLC) and small cell lung cancer(SCLC).In Pulmonary lymphoepithelioma-like carcinoma, EBV was significantly associated with cancer risk, Non-small cell lung cancer (OR = 5.58, 95% CI: 1.98–15.72, P < 0.001, I^2 ^= 79.9%) compared with Small cell lung cancer (OR = 2.13, 95%CI: 0.44–10.26, P = 0.001; I^2 ^= 72.7%) was more significantly associated with EBV; the prevalence of EBV in Non-small cell lung cancer, Small cell lung cancer, and Pulmonary lymphoepithelioma-like carcinoma was 32.43%, 21.17% and 81.08%.

**Table 2:** Summary of ten eligible studies comparing patients with non-small cell lung cancer and small cell lung cancer and lymphoepithelioma-like carcinoma of the lung with non-cancer controls with/without EBV infection.

permutation	study	NSCLC(n=555)		SCLC(n=137)		LELC(n=37)		Control(n=523)		
		EBV(+)	EBV(-)	EBV(+)	EBV(-)	EBV(+)	EBV(-)	EBV(+)	EBV(-)	
1	Jose Javier Gómez-Román (2009)	12	2			0	5	0	90	*In situ* hybridization technique for chromogenic
2	A J Han (2000)	0	17	0	2	30	2	0	19	*In situ* hybridization technique for chromogenic
3	Mahdi Karimi-Shahri (2013)	5	31	0	12			2	40	Polymerase chain reaction (PCR)
4	Xia He Shun (2000)	24	19	0	2			15	17	Polymerase chain reaction (PCR)
5	Wang Mei Mei (2013)	27	52	9	20			1	22	*In situ* hybridization technique for chromogenic
6	Lai Ya Zhou (2005)	12	15	1	2			6	52	Enzyme-linked immunosorbent assay(ELISA)/Polymerase chain reaction (PCR)
7	Zhang Lei (1996)	25	43	2	3			10	77	*In situ* hybridization technique for chromogenic
8	Ma Li Ren (2009)	57	89	16	57			16	16	Indirect fluorescence immunoassay (IFA)
9	Kheir (2019)	2	86	0	8			0	100	*In situ* hybridization technique for chromogenic
10	Dong Xue Feng (2007)	16	21	1	2			0	40	Polymerase chain reaction (PCR)
Combined volume		**180**	**375**	**29**	**108**	30	7	**50**	**473**	
Prevalence		32.43%		21.17%		81.08%		9.56%		

### 3.4 Sensitivity analysis and publication bias

To assess the effect of each study on the pooled OR, we sequentially removed individual studies from the meta-analysis. The pooled OR was stable and showed statistical significance using a random-effects model before and after deletion of either study (data not shown). These data collectively suggest that the results of this meta-analysis are robust and not unduly influenced by any of the fourteen studies.

**Figure 3:**
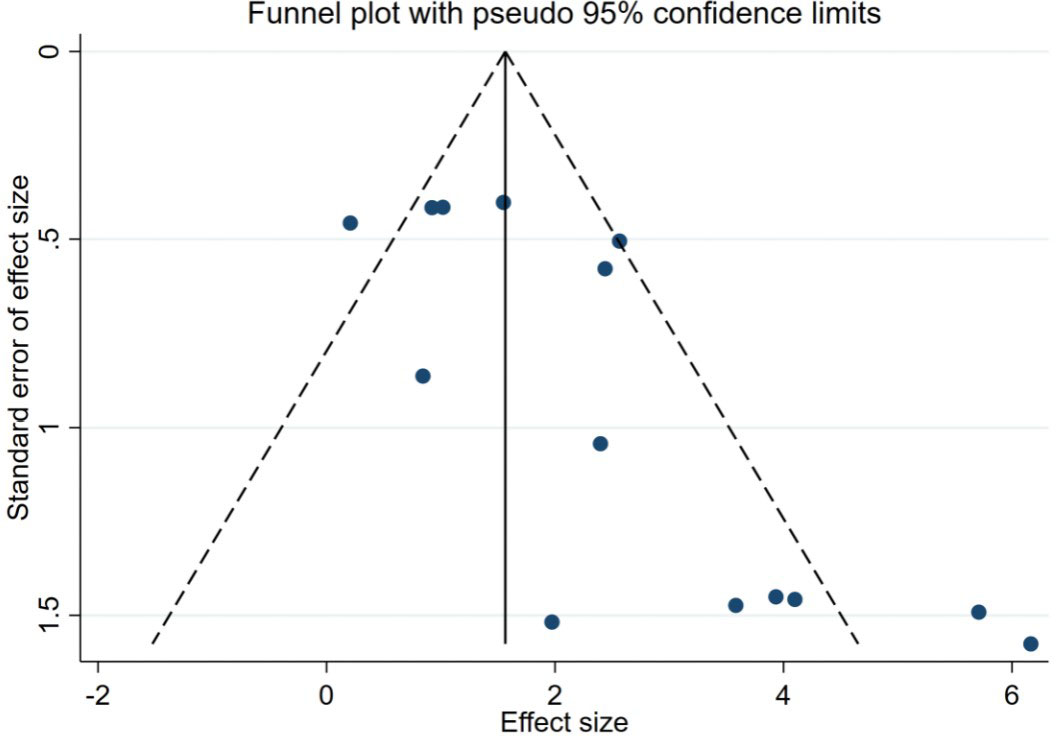
Funnel plot of publication bias in correlation between EBV infection and LC risk.

**Figure 3**, the funnel plot showed some asymmetry, but Begg’s test showed that there was no significant publication bias in this meta-analysis (P =0.08). However, The results of the egger test showed the existence of publication bias (P = 0.007). Furthermore, the limited number of studies (n = 14) suggested potential publication bias.

## 4 Discuss

For the past few decades, the connection between Epstein-Barr virus and lung cancer has been controversy. This controversy may be related to geography, gender, race, sample selection (14), and a broader perspective suggests that the discrepancies regarding the link between Epstein-Barr virus and lung cancer may be attributed to variations in the methods or techniques used to detect EBV in lung cancer samples (18). This is because various detection methods exhibit varying degrees of sensitivity when identifying “EBV positive” diagnoses, and different criteria may be utilized for such identification. *In-situ* Hybridization (EBER-ISH) is a highly sensitive and specific method for visualizing the EBV genome or its transcripts in cells. As a result, it can be employed to differentiate EBV in tumor cells from that in adjacent lymphocytes and detect latent EBV in paraffin sections (1). Nevertheless, studies have shown that EBV gene products, including various EBERs, may not be detectable in EBV-positive tumor cells (20), because the targeted products are inconsistently expressed during latent EBV infection or at certain stages of infection. Moreover, there is the possibility of EBER-negative forms of EBV infection, which could result in false-negative outcomes (21). The Polymerase Chain Reaction (PCR) is a method that has the potential to be an important technique for detecting Epstein-Barr virus (EBV) DNA. However, in the study by Amir Hossein Jafarian et al., two nonneoplastic lung tissue samples from 42 controls showed EBV genome by PCR (4.7%) (17). The authors proposed that the reason for false positives in PCR investigations of non-tumor-infected cells in the control group could be attributed to the prevalence of viruses like EBV that infect a considerable proportion of the population (17). Immunohistochemistry has been utilized for detecting viral proteins, but certain viral proteins exhibit inconsistent expression during latent EBV infection (22). Indirect immunofluorescence (IIF) quantifies antibodies to various antigens in EBV-infected cells: EB nuclear antigen expressed during latency, early antigen expressed during the early lytic cycle, VCA expressed during the late lytic cycle, and membrane antigen expressed on the surface of cells in the late lytic cycle (1). It is unclear whether some EBVs switch from latency to lysis in lung cancer (1). The choice of statistics in our study investigating the detection of EBV DNA using PCR, indirect immunofluorescence (IIF) measures, and ISH techniques, could have contributed to the bias observed in our results.

Our research findings revealed that lung cancer (LC) tissues were more likely to test positive for EBV infection than normal or non-cancerous lung tissues. This includes tissues from adjacent and non-adjacent normal and non-cancer controls (OR = 8.30, 95% CI: 4.08–16.9, P < 0.001; I² = 70.7%). These results provide the most convincing evidence of an association between EBV infection and LC. Our meta-analysis strongly suggests that EBV infection is significantly associated with an increased risk of LC in strict case-control settings, particularly compared to non-cancer controls.

In a total of fourteen literature reviews, we found that the overall prevalence of EBV infection in lung cancer patients was 44.36% (95% CI: 4.08-16.9), supporting the hypothesis that EBV infection is a risk factor for lung cancer. Additionally, EBV infection resulted in a 4.24-fold increased risk of developing lung cancer compared to controls.

Our study revealed a significant difference in the prevalence of EBV infection in lung cancer tissues between China and other regions (odds ratio OR = 9.36, 95% confidence interval CI: 4.00–21.94, P < 0.001, I² = 73.5%). This suggests that EBV infection in China is more strongly associated with lung cancer cases compared to other regions. Furthermore, Begin et al. reported a strong association between EBV infection and lymphoepithelioma-like carcinoma (LELC) in Asian populations (5). According to J. Han et al., EBV infection has a stronger correlation with lymphoepithelioma-like carcinoma (LELC) in southern China (1). The reasons for the observed racial and regional variation in EBV infection prevalence are not yet clear. However, it is possible that this variation is related to differences in EBV prevalence in China and variations in detection methods and diagnostic criteria for EBV infection in other regions. Additionally, the effects of multicultural societies may also be a contributing factor in explaining this difference.

We collected information on the prevalence of EBV in different types of lung cancer, the prevalence of Pulmonary lymphoepithelioma-like carcinoma was 81.08%, the prevalence of Non-small cell lung cancer was 34.78%, and the prevalence of Small cell lung cancer was 21.17%. The statistical analysis revealed that EBV infection was most strongly associated with cancer risk in pulmonary lymphoepithelioma-like carcinoma (LELC). Additionally, the EBV-related risk was significantly higher in non-small cell lung cancer than in small cell lung cancer. Since the association between lung cancer and EBV infection is related to lymphocytic infiltration, this could explain why pulmonary lymphoepithelioma-like carcinoma (LELC) is the type of lung cancer that is most strongly associated with EBV cancer risk. Jose Javier Gómez-Román et al. identified 19 cases of EBV-positive lung adenocarcinoma and squamous cell carcinoma out of 1545 lung tumors. However, 5 of these carcinomas were consistent with lymphoepithelioma-like lung cancer, characterized by prominent lymphoid infiltration associated with EBV infection. Among the remaining cases, 6 were classified as squamous cell carcinomas and 8 were classified as adenocarcinomas. Notably, all 19 EBV-positive lung cancers exhibited a significant inflammatory lymphoid stroma, which gave the tumors a hypoproliferative appearance of lymphocytes (16). In 1994, Kasai conducted a study on Japanese patients with lung cancer. The study included 81 cases and utilized a highly sensitive *in situ* hybridization (ISH) method. Kasai employed antisense oligonucleotide probes to EBV-encoded small ribonucleic acid-1 (RNA-1) to detect the presence of Prestan-Barr early ribonucleoprotein 1 (EBER1) (23). The expression of EBER1 was detected in the study as follows: one case of poorly differentiated squamous cell carcinoma associated with prominent lymphoid stroma, two cases of well-differentiated adenocarcinomas, and two cases of moderately differentiated squamous cell carcinomas (24). The findings of the study showed that the majority of cancer cells in poorly differentiated squamous cell carcinomas exhibited robust EBER1 signaling (23). Wang Meimei and colleagues conducted a study on seventy-seven cases of squamous cell carcinoma and adenocarcinoma. The results showed that the positive rates of EBER-1 were 8.3% (1/12), 47.2% (17/36), and 27.6% for well-, moderately, and poorly differentiated carcinomas, respectively. Furthermore, as the severity of EBV infection increased, the infiltration of lymphocytes also increased, suggesting a correlation between EBV infection and the degree of differentiation in lung cancer tissues, with mainly medium and low differentiation observed (2). Zhang Lei and colleagues investigated eighty-seven cases of non-LELC lung tumors. The results indicated a significant difference in the positive rate of EBV between moderately and poorly differentiated cancers and well-differentiated cancers. Additionally, positive signals were predominantly found in squamous cell carcinoma nests in the lungs based on morphology. Notably, all strongly positive cases observed in peripheral poorly differentiated cells were associated with poorly differentiated tumors (13). Interestingly, in the study by Sun Yali et al., it was pointed out that EB virus positive patients had a higher proportion of adenocarcinoma and lymph node metastasis (8), which may be related to the fact that lung adenocarcinoma is more prone to lymph node metastasis than squamous cell carcinoma.

It is common for the Epstein-Barr virus to infect the lungs, yet the manner in which the virus causes lung cancer remains unknown (2). Several studies indicate that specific genetic factors may be involved in the mechanism by which the Epstein-Barr virus infects lung cells and leads to the development of lung cancer. Han Ajie and colleagues demonstrated that LMP1, a crucial protein for tumor transformation, has the ability to suppress epithelial differentiation and alter the epithelial phenotype (1). Ao Yazhou and colleagues suggested that EBNA-mediated carcinogenesis may influence the regulation of cells and viruses by establishing transcriptional or post-transcriptional connections. Moreover, LMPI can impact apoptosis occurrence by affecting the p53 gene, thus exerting an effect on cell death through multiple pathways. This disturbance of normal cellular metabolism can trigger malignant transformation (13). Xia Heshun and colleagues discovered a significant difference in the expression of Bcl-2 between EBV DNA-positive and EBV DNA-negative patients, implying a potential association between EBV infection and elevated Bcl-2 expression (11). At the same time, it is also pointed out that the proto-oncogene C-myc plays a role in both cell growth and apoptosis (11). Jiang Bo and colleagues’ research findings revealed an up-regulation in miR-BART5 expression within Epstein-Barr virus-positive lung cancer tissues, indicating a potential involvement of miR-BART5 in the initiation and progression of lung cancer via negative regulation of the PUMA gene expression (12). Giovanna E. Carpagnano et al. demonstrated that EBV infection is detectable in EBC and found high EBV positivity in lung cancer patients (19). This may become a suitable non-invasive tool for lung cancer screening and early diagnosis.

## 5 Limitations

This meta-analysis aimed to establish the relationship between EBV infection and lung cancer risk. However, there are a few limitations that should be acknowledged. Firstly, the overall and subgroup analyses demonstrated heterogeneity, leading to some degree of uncertainty in the conclusions that should be considered when interpreting the results. Secondly, all studies included in this analysis were published in English or Chinese, which may introduce language bias, although previous research indicates that this may not significantly affect the results. Thirdly, there are potential concerns regarding the risk of bias. As all studies were retrospective, the completeness of the original data and potential recall bias may have influenced the results. Fourthly, the included studies were small case-control studies with limited sample sizes, reducing the precision of the results. Fifthly, some studies lacked detailed information on the collection of control specimens. Finally, residual confounding may arise due to some studies’ failure to account for confounding variables such as gender, age, marital status, smoking, alcohol consumption, socioeconomic status, and lifestyle.

In summary, the findings of this meta-analysis demonstrate that EBV infection elevates the likelihood of developing lung cancer. Future investigations should prioritize examining the relationship between EBV and various histological subtypes of lung cancer while accounting for more detailed demographic parameters. Such research will provide more dependable insights into the mechanism of lung cancer and facilitate the development of effective programs for lung cancer prevention, diagnosis, and treatment.

## Data availability statement

The original contributions presented in the study are included in the article/supplementary material, further inquiries can be directed to the corresponding author.

## Author contributions

YC, MD and TL contributed to the concept and design of the study. ZX organizes the database. YC performed statistical analysis. YC wrote the first draft of the manuscript. YC, MD, TL and ZX wrote parts of the manuscript. All authors participated in manuscript revision, read and approved the submitted version.
